# Effects of Exercise-Induced ROS on the Pathophysiological Functions of Skeletal Muscle

**DOI:** 10.1155/2021/3846122

**Published:** 2021-10-01

**Authors:** Fan Wang, Xin Wang, Yiping Liu, Zhenghong Zhang

**Affiliations:** Provincial University Key Laboratory of Sport and Health Science, Provincial Key Laboratory for Developmental Biology and Neurosciences, Key Laboratory of Optoelectronic Science and Technology for Medicine of Ministry of Education, School of Physical Education and Sport Sciences, Fujian Normal University, Fuzhou 350007, China

## Abstract

Oxidative stress is the imbalance of the redox system in the body, which produces excessive reactive oxygen species, leads to multiple cellular damages, and closely relates to some pathological conditions, such as insulin resistance and inflammation. Meanwhile, exercise as an external stimulus of oxidative stress causes the changes of pathophysiological functions in the tissues and organs, including skeletal muscle. Exercise-induced oxidative stress is considered to have different effects on the structure and function of skeletal muscle. Long-term regular or moderate exercise-induced oxidative stress is closely related to the formation of muscle adaptation, while excessive free radicals produced by strenuous or acute exercise can cause muscle oxidative stress fatigue and damage, which impacts exercise capacity and damages the body's health. The present review systematically summarizes the relationship between exercise-induced oxidative stress and the adaptions, damage, and fatigue in skeletal muscle, in order to clarify the effects of exercise-induced oxidative stress on the pathophysiological functions of skeletal muscle.

## 1. Introduction

**Figure 1:**
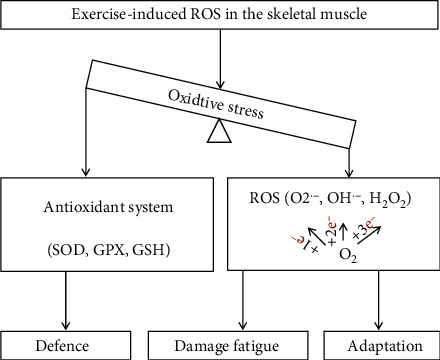
Effects of exercise-induced ROS on the balance of oxidative stress in the skeletal muscle. Exercise-induced oxidative stress occurs as the production of ROS generated in the body, which is excessive to the defense ability of antioxidant system during physical exercise. ROS includes O_2_^−^, OH^−^, and H_2_O_2_, which affect the adaption, damage, and fatigue in the skeletal muscle.

Exercise as a common stressor leads to oxygen supply that cannot meet a rapid increase in oxygen demand of the body; then, many tissues and organs produce some highly active molecules, such as reactive nitrogen species (RNS) and reactive oxygen species (ROS). ROS plays a pivotal role in the stress process, including superoxide anion (O_2_^·-^), hydroxyl radical (OH^·-^), and hydrogen peroxide (H_2_O_2_) (Figure 1) [1]. The production of ROS exceeds its scavenging capacity, which is the reaction of oxidative stress. Under normal physiological conditions, ROS participates in a number of cell activities, including cell energy metabolism, signal transduction, and gene expression regulation, but high levels of ROS can also damage biomacromolecules in cells, such as lipids, proteins, and nucleic acids, leading to cell senescence even death [2]. A large number of studies have shown that regular or suitable exercise produces low-level ROS, while excessive production of endogenous free radicals during exercise can damage the physiological functions of the entire tissue [1–3], such as skeletal muscle [3]. Skeletal muscle is the dynamic part of the exercise system, and the physiological level ROS is an essential substance for maintaining its function, which is involved in the production of muscle force, the maintenance of muscle content, intracellular signal transduction, gene expression, and other related activities, while excessive ROS causes the dysfunction of contraction and muscle weakness [4]. As is well known, ROS induced by skeletal muscle contraction during exercise can increase the level of oxidative stress and enhance the antioxidant defense system. Although exercise-induced reactive oxygen species are required for normal force production in skeletal muscle, the high levels of ROS can contribute to contractile dysfunction [5]. The sarcoplasmic reticulum Ca^2+^ release channel is highly sensitive to ROS, which will reduce the sensitivity of myofibrils to Ca^2+^ and then affect muscle contraction [5, 6]. In addition, the accumulation of ROS in the body depends on exercise mode, exercise intensity, and duration. Therefore, exercise-induced oxidative stress may play an important role in the pathophysiological functions of skeletal muscle.

## 2. Exercise-Induced Oxidative Stress

In 1978, exercise-induced oxidative stress was firstly proposed, which refers to oxidative stress caused by exercise; that is, the rate of free radicals generated in the body is much greater than its scavenging rate during physical exercise, which will damage tissue, resulting in the decline of the body's working ability [7]. The mitochondrial respiratory chain is the main source of endogenous ROS during exercise, and its excessive production can cause oxidative damage to lipids, proteins, nucleic acids, and other substances [8]. The accumulation of ROS in the body depends on the exercise mode, intensity, and duration. Studies have indicated that a small amount of ROS produced by moderate-intensity exercise can act as a second messenger in cells to mediate growth factor signal transmission [9–11]. Acute exercise can promote the excessive production of ROS, which causes an imbalance in the oxidation-antioxidant homeostasis in cells, because rapidly increasing oxygen consumption inevitably produces more free radicals during strenuous exercise [12]. A large amount of oxygen is consumed to produce a large amount of singlet oxygen (O_2_^·-^), which stimulates a series of radical chain reactions [13]. On the other hand, the hypoxia of local tissue and the accumulation of metabolites affect the mitochondrial energy supply. Meanwhile, high-intensity exhaustive exercise can cause a significant increase in the content of malondialdehyde (MDA) and further increase with the extension exercise [14]. MDA can objectively reflect the level of free radicals in the body.

## 3. ROS in Skeletal Muscle

Skeletal muscle is an important exercise organ in the body; its normal physiological function is the basic prerequisite and important guarantee for health.

### 3.1. ROS in Different Skeletal Muscle Fibers

Skeletal muscle is composed of three types of muscle fibers: I, IIa, and IIb. The abilities of these different types of muscle fibers to generate ROS and resist oxidative stress are also different [15]. The leakage level of mitochondrial ROS in type IIb muscle fiber is 2-3 times that of type IIa muscle fiber, due to mitochondria in type IIb muscle fiber playing an important role in the production and release of superoxide [4]. High-intensity exercise leads to the excessive production of ROS, which can cause changes in the normal physiological environment of skeletal muscle fibers and vascular endothelial dysfunction. The levels of antioxidant enzymes of type I and IIa muscle fibers enhance the scavenging ability of free radicals and promote the recovery of skeletal muscle motor functions, while there is no significant change in type IIb muscle fibers. In addition, type I muscle fibers are rich in myoglobin with the strongest abilities of aerobic metabolism and antifatigue [16]. These findings indicate that different types of muscle fibers have different effects on antifatigue and ROS production, but they are all related to their antioxidant capacity.

### 3.2. Sites for ROS in Skeletal Muscle Cells

In the past, mitochondria are the main site of intracellular ROS in contracting skeletal muscle [14]. In mitochondria, 2–5% of the total oxygen consumed may undergo one electron reduction with the generation of superoxide [17]. Mitochondrial complex I in the electron transport chain releases superoxide to the mitochondrial matrix, while complex III to both sides of the inner membrane [18]. Recently, some scholars have discovered that the main source of ROS may not be limited to mitochondria during exercise [14]. The upper limit of the total utilization of oxygen consumed by mitochondria in different tissues to produce ROS is about 0.15% [19]. Simultaneously, a large number of studies have shown that many intracellular enzymes are involved in the production of ROS, such as NADPH oxidases (NOXs), xanthine oxidase (XO), and phospholipase A2 (PLA2) [18]. Regardless of whether muscle is in a resting or contracting state, NOX family proteins can produce more superoxide anions than that of mitochondria in single muscle fibers [20]. Moreover, the NOX2 loss-of-function model indicated that NOX2 may be the main source of cytosolic ROS in skeletal muscle during moderate-intensity exercise [21]. Another study found that there was a correlation between XO content and lactic acid level during anaerobic exercise, producing a large amount of ROS, thereby aggravating skeletal muscle oxidative damage [22]. In addition, muscle damage caused by exercise can also stimulate various cytokines to activate macrophages and neutrophils, leading to the overproduction of ROS [23].

## 4. Skeletal Muscle Adaptation

**Figure 2:**
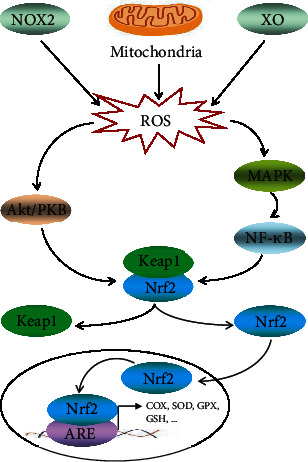
Effects of exercise-induced ROS on the signaling pathway in the skeletal muscle. Exercise-induced ROS can activate MAPK and Akt signaling pathways. Nrf2 dissociates from its cytoplasmic inhibitor Keap1 then translocates to the nucleus and combines with ARE to contribute to some downstream antioxidant gene transactivation, such as the oxidases, COX, SOD, GPX, and GSH.

Skeletal muscle is a highly plastic tissue. Moderate or regular exercise training can regulate the signaling pathways by oxidative stress and ROS to resist oxidative damage and further come true structure-function adaptations in skeletal muscle [19, 24, 25]. ROS can not only activate NF-*κ*B by activating mitogen-activated protein kinase (MAPK) but also stimulate Akt phosphorylation, thereby activating their common downstream molecule nuclear factor erythroid-derived 2-related factor 2 (Nrf2) [25]. Nrf2 as a redox-sensing transcription factor dissociates from its cytoplasmic inhibitor Keap1, then translocates to the nucleus and combines with antioxidant response element (ARE) to contribute to transactivation of some downstream antioxidant genes, especially defense-related enzymes and exercise-adapting-related enzymes, such as oxidase cytochrome oxidase (COX), superoxide dismutase (SOD), glutathione peroxidase (GPX) activities, and glutathione (GSH), thereby upregulating their expressions and activities, alleviating the oxidative damage, and promoting exercise-induced adaptations (Figure 2) [19, 24, 25]. Acute exercise or long-term exercise training may increase the expression of Nrf2 and/or Nrf2-ARE binding activity. After 6 weeks of treadmill training, the transcription activity of Nrf2 in skeletal muscle is increased, and the activity of COX is also increased by 20% [26].

Moreover, exercise-activated Nrf2 may regulate skeletal muscle mitochondria by promoting mitochondrial biogenesis, improving mitochondrial respiratory function, and regulating mitochondrial autophagy. In addition, peroxisome proliferator-activated receptor-*γ* coactivator-1*α* (PGC-1*α*) plays an important role in glucose uptake, mitochondriogenesis, and hypertrophy in skeletal muscle responses to physical exercise [24]. Exercise-induced ROS can couple with lactate metabolism to stimulate PGC-1*α* production, especially endurance exercise [24]. PGC-1*α* coactivates nuclear respiratory factor- (NRF-) 1 and NRF-2 to increase their DNA binding in skeletal muscle after acute exercise [24], then activates the genes encoding COX and mitochondrial transcription factor A (TFAM), which activate mitochondrial DNA transcription, thereby increasing mitochondrial synthesis [24]. In addition, exercise-induced ROS are able to stimulate the skeletal muscle to secrete myokines, which also play important roles in the regulation of cell signaling and muscle metabolic adaptation [27, 28], such as interleukin-15 (IL-15). IL-15 is a regulator to control intracellular ROS production and attenuate oxidative stress in skeletal muscle cells [29].

## 5. Skeletal Muscle Cell Damage

Exhaustive exercise can increase the peroxidation and weaken the antioxidant capacity in skeletal muscles, which can cause the damage of skeletal muscle cells through modification of lipid, protein, and DNA.

Firstly, for lipid damage, biomembrane is the main component of cells. Lipid peroxidation caused by excessive endogenous ROS during exercise can change the liquidity, fluidity, and permeability of the biomembrane, which in turn leads to membrane dysfunction [30]. The lipid bilayer contains a large amount of polyunsaturated fatty acid (PUFA). ROS can deprive the hydrogen at the diallyl position in PUFA to form unsaturated fatty acid free radicals, then combine with oxygen molecules to generate lipid peroxyl radicals LOO^·-^ [31]. LOO^·-^ can deprive the allyl groups of unsaturated fatty acid hydrogen which forms a new unsaturated fatty acid free radical and lipid peroxide (LOOH), causing oxidative damage to the biomembranes of skeletal muscle cells [31].

Secondly, for protein damage, ROS plays a very important role in the process of protein metabolism, which can promote protein turnover and renewal under physiological conditions. But excessive ROS induced by exercise will cause damage to proteins and induce the inactivation of enzymes, the loss of receptors, and the decline of immune function [31]. The damage of proteins by ROS is mainly to change their activities. OH^·-^ can change the primary structure of the protein and contribute to the secondary and tertiary structure modification, so that the polypeptide chain unfolds to form a random structure [32]. These modifications expose the originally shielded peptide bonds to proteolytic enzymes. In addition, a protein is combined with OH^·-^ which will increase the production of double tyrosine, further induce the breakage, which is manifested by the effect of extracting hydrogen on the a-carbon atom of the amino acid, and then react with O_2_^·-^ to generate LOO^−^, ultimately forming peroxides [32].

The last is DNA damage. 8-Hydroxydeoxyguanosine (8-OHdG) is an important marker for the detection of oxidative DNA damage. The C-8 position of the base guanine in the DNA chain is susceptible to the attack of OH^·-^ and O_2_^·-^ and undergoes hydroxylation to form the adduct 8-OHdG [33]. OH^−^ combines with the deoxyguanine nucleotide residues at the C-4, C-5, or C-8 positions of DNA bases to form 8-hydroxy-7,8-dihydroxyguanine nucleotides, which in turn further oxidizes to generate 8-hydroxydeoxyguanine nucleotides. Simultaneously, during the replication process, 8-OHdG on the DNA chain can pair with other bases than C to form point mutations, such as GC→TA [32].

## 6. Skeletal Muscle Fatigue

The process of sports fatigue is a complex physiological phenomenon; it actually occurs with the consumption of physical energy or the accumulation of metabolites, including skeletal muscle fatigue, visceral fatigue, and nerve fatigue. Skeletal muscle fatigue is the main peripheral manifestation of sports fatigue. The production of skeletal muscle strength depends on its contraction mechanisms, while any disorder of the nerves, ions, blood vessels, and energy systems upstream of the cross bridge will lead to their loss and cause muscle fatigue, especially the energy metabolism factors during the process of muscle contraction, such as H^+^, lactic acid, Pi, reactive ROS, heat shock protein (HSP), *α*-acid glycoprotein (ORM) which also affect muscle fatigue.

Excessive ROS produced by strenuous exercise may cause exercise fatigue by reducing the release of skeletal muscle sarcoplasmic reticulum Ca^2+^ and/or the sensitivity of myofibrils Ca^2+^ [34]. However, under mild oxidative stress induced by physical exercise, the S-glutathionization of troponin can improve contractile apparatus Ca^2+^ sensitivity in fast-twitch fibers, thereby delaying the occurrence of fatigue [35]. However, antioxidants restored Ca^2+^ to release in the sarcoplasmic reticulum, but the decrease in muscle strength during fatigue cannot be eliminated [36], indicating that oxidative stress caused by exercise is closely related to muscle fatigue. Endogenous and exogenous ROS not only may hinder muscle fiber contraction by affecting the release and uptake of Ca^2+^ in the sarcoplasmic reticulum and reducing the activity of troponin but also can promote the occurrence of exercise fatigue by destroying mitochondrial functions and inhibiting aerobic metabolism [6]. In addition, during exercise, the increased energy metabolism rate leads to excessive mitochondrial ROS production in fatigued skeletal muscle cells, which further leads to the occurrence of the oxidative modification of lipids, proteins, and DNA.

## 7. Conclusion

Together, exercise-induced oxidative stress has dual effects on skeletal muscle tissue. Proper exercise can promote generation of the physiological levels of ROS, maintain the normal skeletal muscle function, and facilitate exercise adaptation, while exhaustive exercise can cause the body to produce too much ROS, which causes excessive oxidative stress, leading to fatigue and cell damage of skeletal muscle. Therefore, further understanding the effect of exercise-induced oxidative stress on the pathophysiological functions of skeletal muscle is very important for guiding exercise to promote health and provides a direction to explore the molecular mechanism of exercise to improve the development of pathophysiological processes in oxidative stress-related diseases.

## Data Availability

The original contributions presented in the study are included in the article. Further inquiries can be directed to the corresponding authors.
